# Two proteins co‐expressed with photosynthetic regulators affect photosynthetic dynamics linked to changes in thylakoid pigment content and composition

**DOI:** 10.1111/plb.70266

**Published:** 2026-07-20

**Authors:** J.‐M. Keller, F. Dinc, E. Kaiser, A. Zupok, B. A. Morris, T. von Bismarck, V. Correa Galvis, W. Thiele, D. M. Kramer, P. Jahns, M. A. Schöttler, U. Armbruster

**Affiliations:** ^1^ Max Planck Institute of Molecular Plant Physiology, Science Park Potsdam Germany; ^2^ Jan Ingenhousz Institute Wageningen Netherlands; ^3^ Department of Biochemistry and Molecular Biology Michigan State University East Lansing Michigan USA; ^4^ Plant Biochemistry Heinrich‐Heine‐University Düsseldorf Düsseldorf Germany; ^5^ Biology Heinrich Heine University Düsseldorf Düsseldorf Germany

**Keywords:** Chlorophyll content, NPQ dynamics, regulation of photosynthesis

## Abstract

- Rapid fluctuations in light intensity require swift regulatory adjustments of photosynthesis. Although the major plant regulators of fast photosynthetic responses have been identified, additional genes with conditional or subtle effects are likely still undiscovered. We hypothesized that genes co‐expressed with the core regulators *PGR5*, *KEA3*, *STN7* and *ZEP* also participate in photosynthetic regulation.
- Transcriptome co‐expression analysis defined a set of ‘*CERP*’ (*CO‐EXPRESSED‐WITH‐REGULATION‐OF‐PHOTOSYNTHESIS*) candidate genes. *Arabidopsis thaliana* T‐DNA insertion mutants for selected *CERP* loci were isolated. Spectroscopic measurements of whole plants and isolated thylakoid membranes, pigment analyses, native/denaturing gel electrophoresis coupled with immunoblotting and fluorescence microscopy were used to assess photosynthetic parameters under constant light and under rapidly fluctuating light regimes and determine photosynthetic complex composition, as well as localize the proteins.
- Two mutants showed alterations in non‐photochemical quenching (NPQ) during light fluctuations. Mutants lacking *CERP1,* also known as *AAF,* showed delayed NPQ relaxation after high‐to‐low light transitions. Mutants lacking *CERP4b*, also known as *FLAP1*, exhibited a higher NPQ during high light phases. These NPQ phenotypes were linked to changes in leaf pigment content and composition. Further characterization of CERP4b/FLAP1 points towards a function of this protein in the formation or composition of the PSII supercomplex.
- Co‐expression mining successfully uncovered additional components that contribute to rapid photoprotective acclimation. CERP1/AAF is essential for fast NPQ relaxation, while CERP4b/FLAP1 contributes to NPQ in high light, likely through effects on PSII antenna. These findings broaden the known regulatory network governing NPQ by including two components that modulate leaf pigment content and composition.

## Graphical abstract

Two factors identified by co‐expression analysis of photosynthetic regulators are described, which are involved in thylakoid pigment content and composition and, through their function, shape the dynamics of NPQ.

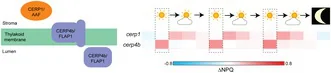

## INTRODUCTION

In nature, plants must frequently adjust to environmental fluctuations. The factor that changes most rapidly is light intensity. Most plants perform well even under strongly fluctuating light intensities by engaging a multitude of regulatory mechanisms that act on various time scales (reviewed by Kaiser *et al*. 2019).

Rates of photosynthetic reactions are directly impacted by light intensity. When light intensity rises rapidly, the carbon‐fixation reactions become limiting for the overall rate of photosynthesis. Consequently, non‐photochemical quenching (NPQ) is activated, leading to the dissipation of excess absorbed light energy as heat (reviewed by Müller *et al*. 2001). Multiple components contribute to NPQ, with energy‐dependent quenching (qE) accounting for the largest part in land plants. qE is also the most rapidly reversible NPQ mechanism, allowing a fast coordination of the light reactions with carbon fixation rates (von Bismarck *et al*. 2023). qE is activated by thylakoid lumen acidification, resulting in protonation of PsbS and the activation of the lumen‐located enzyme violaxanthin de‐epoxidase (VDE), which converts violaxanthin into zeaxanthin (Zx; Briantais *et al*. 1979; Demmig‐Adams *et al*. 1990; Li *et al*. 2000; Li *et al*. 2002). Zx is bound by light harvesting antenna proteins of the PSII supercomplex, which upon protonation of the PsbS protein undergo a conformational rearrangement that facilitates the thermal dissipation of absorbed light energy (Correa‐Galvis *et al*. 2016; Sacharz *et al*. 2017; Nicol *et al*. 2019; Wilson *et al*. 2024). The largest PSII supercomplex (C_2_S_2_M_2_) contains the PSII dimer (C_2_), two strongly (S_2_) and two moderately (M_2_) bound LHCII trimers as well as the PSII minor antenna proteins CP24, CP26 and CP29 (Su *et al*. 2017).

Several regulatory mechanisms affect lumen acidification and thereby qE. One of these involves the thylakoid localized PGR5 protein (Munekage *et al*. 2002). Plants lacking this protein die in strongly fluctuating light conditions, because they cannot sufficiently build up the proton motive force across the thylakoid membrane to fully induce qE and down‐regulate linear electron transport by ‘photosynthetic control’ at the cytochrome b_6_f complex, resulting in a massive acceptor side limitation of PSI (Suorsa *et al*. 2012). Another factor that facilitates qE relaxation when carbon reactions are no longer limiting is the thylakoid K^+^/H^+^ exchange protein KEA3 (Armbruster *et al*. 2014; von Bismarck *et al*. 2023). In addition, qE dynamics are modulated by xanthophyll cycling, especially the balance between VDE activity and that of the antagonistic zeaxanthin epoxidase (ZEP), which is associated with the stromal surface of the thylakoids (Niyogi *et al*. 1998; Bethmann *et al*. 2023).

A stronger excitation of photosystem II (PSII) than photosystem I (PSI) leads to a process called state transitions, during which the light‐harvesting complex of PSII (LHCII) is phosphorylated by the thylakoid protein kinase STN7, detaches from PSII and migrates to PSI (reviewed by Goldschmidt‐Clermont & Bassi 2015; Rochaix 2007). The migration of LHCII away from PSII also contributes to NPQ, but at much lower rates than qE, at least in land plants (Krause & Weis 1991; Bellafiore *et al*. 2005). STN7 is activated by a reduced redox state of the plastoquinone pool, signalling excess activity of PSII relative to PSI (Bellafiore *et al*. 2005; Goldschmidt‐Clermont & Bassi 2015).

While most of the regulatory factors with strong effects on NPQ under steady‐state conditions were described within the last 25 years by use of forward and reverse genetics, the details of the underlying molecular mechanisms are still under debate. It is likely that proteins with conditional functions and/or smaller effects on NPQ remain undescribed. For example, the discovery that thylakoid ion‐transport proteins facilitate qE dynamics was made only 10–12 years ago (Armbruster *et al*. 2014; Duan *et al*. 2016; Herdean *et al*. 2016), many years after the molecular natures of the other regulatory factors with steady state NPQ effects were described (Niyogi *et al*. 1998; Li *et al*. 2000; Munekage *et al*. 2002; Bellafiore *et al*. 2005).

Despite the ambitious goal to describe the function of all genes of the model plant *Arabidopsis thaliana* (Arabidopsis) by 2010 (Somerville & Dangl 2000), which is now 16 years past, a large number of its genes remain uncharacterized. Many Arabidopsis genes are conserved across plants and green algae (Karpowicz *et al*. 2011), suggesting that they evolved together with photosynthesis or related metabolic processes. Encoded proteins often localize to the chloroplast, further supporting a function in photosynthesis or other metabolic processes specific for this organelle.

Previously, it was shown that Arabidopsis mutants with suspected defects in photosynthesis showed wild‐type (WT)‐like photosynthetic phenotypes when grown in constant light throughout the day. However, under variable light conditions, differences from WT phenotype became evident in photosynthetic parameters, such as NPQ (Cruz *et al*. 2016).

We found that the photosynthetic regulators PGR5, KEA3, STN7 and ZEP are tightly co‐regulated at the transcript level and hypothesized that genes co‐expressed with this network might likewise play a role in photosynthetic regulation. We analysed Arabidopsis T‐DNA insertion mutants of these C*O‐EXPRESSED‐WITH‐REGULATION‐OF‐PHOTOSYNTHESIS* (*CERP*) genes and found that previously described mutants of two genes differed most strongly in their NPQ responses in fluctuating light. For CERP1, which was previously linked to senescence and called Arabidopsis A‐15 (AAF) (Chen *et al*. 2011, 2012), we describe a key function under fluctuating light conditions. For CERP4b, previously linked to chloroplast proton homeostasis under fluctuating light and therefore named ‘FLUCTUATING‐LIGHT‐ACCLIMATION PROTEIN1’ (FLAP1; Sato *et al*. 2017), we describe a co‐migration with the PSII supercomplex and a likely function in PSII antenna assembly, stability or composition.

## MATERIALS AND METHODS

### *In silico* analyses

Co‐expression analyses were done using ATTED‐II (https://atted.jp; Obayashi *et al*. 2008) and *KEA3* (*AT4G04850*), *ZEP* (*AT5G67030*), *STN7* (*AT1G68830*) and *PGR5* (*AT2G05620*) as queries. The network was drawn using the ATTED‐II NetworkDrawer with display type cytoscape. The transit peptides and transmembrane domains were predicted using TargetP (Armenteros *et al*. 2019) and DeepTMHMM (Hallgren *et al*. 2022), respectively. Sequence alignment was done using ClustalW (https://www.genome.jp/tools‐bin/clustalw) and phylogenetic reconstructions using PhyloGenes (Zhang *et al*. 2020; https://phylogenes.arabidopsis.org).

### Transient expression of C‐terminal YFP fusions in *Nicotiana benthamiana* leaves

The coding sequences of the individual *CERP*s and *YFP* with overlaps for a C‐terminal fusion were amplified and assembled into pEG100 by Gibson assembly to generate *CERP‐YFP* fusions. The *Agrobacterium tumefaciens* (Agrobacterium) strain GV3101 was transformed with the respective constructs. For transient expression in *N. benthamiana*, a culture of the respective Agrobacterium strain was resuspended in induction medium (10 mM MgCl_2_, 10 mM MES‐KOH pH 5.6, 150 μM acetosyringone) to an OD_600_ of 0.5. After 2 h at 28 °C, suspensions were inoculated onto sections of well‐watered *N. benthamiana* leaves by injecting into the bottom side (Blatt & Grefen 2014). Transfected plants were grown for 2 days before leaf discs were analysed for the subcellular localization of the YFP fluorescence signal. For microscopy, the Leica TCS SP5 instrument was used with 63x/1.4 objective and water immersion. Fluorophores were excited by using an argon laser (intensity: 20%) at 514 nm, YFP fluorescence was detected between 524 and 582 nm and chlorophyll (Chl) fluorescence between 618 and 800 nm (gain settings: PMT 5, 660 V; PMT‐Trans, 436 V).

### Plant material and growth conditions

The T‐DNA Arabidopsis (*A. thaliana*) insertion lines were obtained from NASC (Sessions *et al*. 2002; Alonso *et al*. 2003; Woody *et al*. 2007; Kleinboelting *et al*. 2011). The T‐DNA insertions were confirmed by gene and T‐DNA specific primers and characterized by sequencing the flanking regions (Tables S1 and S2). In the case of segregating T2/T3, seed stocks were provided by the stock centre, mutant and WT were selected by PCR from the same seed pool and both genotypes were initially analysed alongside for comparison. For generating *CERP4B* overexpression constructs, the coding region was amplified from cDNA using gene‐specific primers adding a sequence encoding for a C‐terminal MYC‐tag and inserted into the binary vector pEarleyGate100 (pEG100; Earley *et al*. 2006) linearized with *Xba*l and *Xho*l restriction enzymes downstream of the *35S* promoter by using Gibson Assembly (Gibson *et al*. 2009). Primers used for genotyping and cloning can be found in Table S1. Homozygous *cerp4b‐2* plants were transformed by floral‐dip as described previously (Clough & Bent 1998). Transgenic plants were selected based on their resistance to BASTA and the expression of the fusion protein was detected by immunoblot analysis using an antibody against the Myc tag.

Arabidopsis plants were grown in pots on soil (Stender, Schermbeck, Germany) containing 1 g/l Osmocote or on Murashige and Skoog medium with 0.8% agar (w/v) supplemented with 2% sucrose (w/v). When sown in soil, seedlings were grown in long day (16 h light/8 h darkness at 20 °C/6 °C, 60%/75% relative humidity, respectively) at 250 μmol photons m^−2^ s^−1^ for 7 days. Then plants were moved to a growth light of 120–150 μmol photons m^−2^ s^−1^ and 16 °C night temperature with the other environmental factors being kept as before. After 2 weeks, single plants were pricked out from the sowing pots and transferred to individual pots. For comparing the effect of light fluctuations, plants were grown from seeds in control light (CL) with 250 μmol photons m^−2^ s^−1^; high light (HL, 900 μmol photons m^−2^ s^−1^) and fluctuating light (FL) with 90 μmol photons m^−2^ s^−1^ for 4 min, followed by 900 μmol photons m^−2^ s^−1^ for 1 min in 12‐h day/night cycles from the seedling stage to time of analysis. Temperature and humidity were set to 20 °C and 60% during the day and 16 °C and 75% humidity at night.

### Spectroscopic measurements of leaves

Most experiments under light fluctuations used the MAXI IMAGING‐PAM (Walz, Effeltrich, Germany) for Chl *a* fluorescence analysis of the entire rosette. Minimal fluorescence F_0_ and maximal fluorescence F_m_ of 30 min dark‐acclimated plants were measured before and during the application of a 0.8 s saturating light pulse, respectively. The maximum yield of PSII F_v_/F_m_ was calculated as (F_m_‐F_0_)/F_m_. Under light fluctuations, non‐photochemical quenching (NPQ) was determined as (F_m_‐F_m′_)/F_m′_.

For across days analysis with varying light conditions, Chl *a* fluorescence of the entire rosettes was monitored using the DEPI‐chamber as described earlier (Cruz *et al*. 2016) at the Center for Advanced Algal and Plant Phenotyping at Michigan State University (CAAPP).

Light‐response curves and further spectroscopic analyses were performed on single young fully expanded leaves. Chl *a* fluorescence was determined with the fiberoptics version of the DUAL PAM‐100 instrument (Heinz Walz GmbH, Effeltrich, Germany) on single leaves after 30 min of dark acclimation. Light‐response curves of PSI‐related parameters were measured with the plastocyanin‐P_700_ version of the Dual‐PAM instrument (Schöttler *et al*. 2007) and the fractions of PSI reaction centres limited at the acceptor side, Y(NA) and donor side Y(ND) were determined (Schreiber & Klughammer 2016). Plants taken directly from the growth chamber were pre‐illuminated with saturating light for 3 min prior to measurements to activate the Calvin–Benson–Bassham (CBB) cycle and avoid an acceptor‐side limitation of PSI. Then, after 10 s in the dark, the maximum difference absorbance signals of redox‐active plastocyanin and PSI were quantified by far‐red illumination for 8 s, followed by a short saturating light pulse (250 ms duration, 5000 μmol photons m^−2^ s^−1^ intensity). The signal amplitude between the fully reduced state in darkness after the end of the saturating light pulse and the fully oxidized state of both plastocyanin and P_700_ at the beginning of the saturating light pulse was used as a measure for the total amount of redox‐active plastocyanin and P_700_, respectively. To measure the light intensity‐dependent response of respective parameters, the actinic light intensity was stepwise increased with a measuring time of 180 s per light intensity under light‐limited conditions and 60 s under light‐saturated conditions. The thylakoid conductivity for protons (*g*H^+^), a proxy for chloroplast ATP synthase activity, was determined from the rapid first phase (0–250 ms in the dark) of the dark‐interval relaxation kinetics of the electrochromic shift signal (Baker *et al*. 2007)—using a KLAS‐100 spectrophotometer (Rott *et al*. 2011).

### Pigment analyses

For the quantification of Chl only, total Chl was extracted from a 1 cm diameter leaf disc by the addition of 80% (v/v) acetone and measured according to Porra *et al*. (1989). For the quantification of all thylakoid pigments, total Chl, xanthophylls and ß‐carotene were extracted by addition of 100% (v/v) acetone. The supernatant was filtered through a 0.2 μm membrane filter (GE Healthcare, Buckinghamshire, United Kingdom). Pigments were separated and quantified by HPLC analysis as previously described (Färber & Jahns 1998).

### Protein isolation, gel electrophoresis and immunodetection

Total protein was extracted from pulverized leaf material using sample buffer (0.1 M Tris pH 6.8, 24% glycerol (v/v), 8% SDS (w/v), 5% mercapto‐ethanol (v/v), 0.02% bromophenol blue (w/v)) and heating at 40 °C for 30 min. Thylakoid membranes were isolated as described previously (Armbruster *et al*. 2014). For SDS polyacrylamide gel electrophoresis (PAGE), total protein extracts or thylakoid samples incubated with sample buffer were resolved on a 16% (w/v) polyacrylamide gel containing 4 M urea (Schägger 2006). For blue native‐PAGE (BN‐PAGE), thylakoid membranes at a concentration equal to 1 mg of total Chl/mL were solubilized with 1% (w/v) β‐n‐dodecyl‐d‐maltoside (DDM) and separated on a 6–12% (w/v) polyacrylamide gradient gel according to Peng *et al*. (2008). The BN‐PAGE was further fractionated by SDS‐PAGE. For this, BN‐PAGE gel strips were first incubated with 66 mM Na_2_CO_3_, 2% SDS (w/v) and 50 mM DTT at 50 °C for 30 min to denature proteins. After electrophoresis, gels were used for immunoblotting analyses. For immunoblot analyses with specific antibodies, gels were blotted onto nitrocellulose and visualized with Ponceau Red (0.1% Ponceau S (w/v) in 5% (v/v) acetic acid) prior to immunodetection. Membranes were blocked with 50 mM Tris, 10 mM NaCl, 0.05% (v/v) Tween 20, 0.2% (v/v) Triton™ X‐100 and 5% (w/v) nonfat dry milk and incubated with antibodies against PsbE (AS06112), Lhcb1 (AS01004), Lhcb2 (AS01003), Lhcb3 (AS01002), Lhcb4 (AS04045), Lhcb6 (AS01010), RbcL (AS03037), Tic40 (AS10709), Actin (AS214615‐10), Myc (AS153034), all obtained from Agrisera (Sweden) and diluted according to manufacturer's instructions. For immunodetection of CERP1 and CERP4b, specific antibodies were produced by Pineda Antikörper‐Service (Germany; peptide sequences AINALKKQWEVEEGD and QLSIEERGKFDEETL for CERP1 and CERP4b, respectively) and provided as monospecific IgGs purified by affinity chromatography using peptide‐coupled column material. For secondary antibodies coupled to the horseradish peroxidase, a mouse‐ or rabbit‐specific antibody was used from Agrisera (against Actin; AS09627) or Sigma‐Aldrich (A8275), respectively, at a dilution of 1:10,000. Signals were detected using the SuperSignal™ West Femto Chemiluminescence‐substrate (ThermoFisher) and the G‐Box‐ChemiXT4 (Syngene) or the C‐DiGit Blot Scanner (Li‐Cor).

### Chloroplast isolation and fractionation

Intact Arabidopsis chloroplasts were isolated via a two‐step Percoll gradient centrifugation (Kunst 1998). In brief, the homogenized leaf tissue was fractionated on the gradient and intact chloroplasts were collected from the interphase. Chloroplasts were fractionated into envelope, thylakoids and stroma via a three‐step sucrose gradient centrifugation after being hypotonically lysed in 10 mM Tris/HCl, pH 8.0 and 1 mM EDTA, pH 8.0, at a Chl concentration of 2 mg/mL (Li *et al*. 1991).

### Photosynthetic complex quantification

Thylakoid membranes were isolated as previously described (Schöttler *et al*. 2004). The contents of PSII and the Cyt *b*_*6*_ *f* were determined from difference absorbance signals of Cyt *b*_559_ (PSII) and the cytochromes *b*_*6*_ and *f*, respectively. To improve the optical properties of the samples, thylakoids equivalent to 50 μg Chl mL^−1^ were de‐stacked in low‐salt medium supplemented with 0.03% (w/v) DDM (Kirchhoff *et al*. 2002). All cytochromes were fully oxidized by the addition of 1 mM potassium hexacyanoferrate (III). Then, 10 mM sodium ascorbate was added to reduce the high‐potential form of cyt *b*_559_ and cytochrome f. Finally, the addition of 10 mM sodium dithionite reduced the low potential form of cyt *b*_559_ and the two b‐type hemes of cytochrome *b*_6_. Using a V‐750 spectrophotometer equipped with a custom‐made head‐on photomultiplier (Jasco GmbH, Pfungstadt, Germany), 10 absorbance spectra were measured between 575 and 540 nm with a spectral bandwidth of 1 nm and a scanning speed of 100 nm min^−1^ at each of the three redox conditions, and subsequently averaged. Difference spectra were calculated by subtracting the spectrum measured in the presence of hexacyanoferrate from the ascorbate spectrum, and by subtracting the ascorbate spectrum from the spectrum measured in the presence of dithionite, respectively. Finally, a baseline calculated between 540 and 575 nm was subtracted from the signals. Then, the difference spectra were deconvoluted as previously described (Kirchhoff *et al*. 2002). Plastocyanin content, relative to PSI, was determined in intact leaves, and absolute plastocyanin contents were calculated after PSI quantification (Schöttler *et al*. 2007). PSI was quantified from light‐induced difference absorbance changes of P_700_. Thylakoids equivalent to 50 μg Chl mL^−1^ were solubilized in the presence of 0.2% (w/v) DDM. After the addition of 10 mM sodium ascorbate as electron donor and 100 μM methyl viologen as electron acceptor, P_700_ was fully photo‐oxidized by applying a light pulse (250 ms length, 2,000 μmol photons m^−2^ s^−1^ intensity). Measurements were performed with the PC‐P700 version of the Dual‐PAM instrument (Heinz Walz GmbH). Finally, based on the Chl content of the investigated leaves, all complex contents were re‐normalized to a leaf area basis.

### 77 K chlorophyll a fluorescence analysis

A F‐6500 fluorometer (Jasco Inc., Groß‐Umstadt, Germany) was used to measure 77 K chlorophyll‐a fluorescence emission spectra. Freshly isolated thylakoid membranes equivalent to 10 μg chlorophyll ml^−1^ were excited at 430 nm wavelength with a bandwidth of 10 nm, and the emission spectrum was recorded between 655 and 800 nm wavelength in 0.5 nm intervals with a bandwidth of 1 nm.

## RESULTS

### Selected genes co‐regulated on transcript level with genes encoding for regulatory factors of photosynthesis are localized in the chloroplast

We found *KEA3* to be co‐regulated at the transcript level with several key regulators of photosynthetic responses that affect NPQ during light fluctuations (*i.e*., *ZEP*, *STN7* and *PGR5*). Based on this observation, we hypothesized that other genes within the same network, especially those with limited or incomplete functional characterization, might also play roles in the regulatory processes of photosynthesis.

**Fig. 1:**
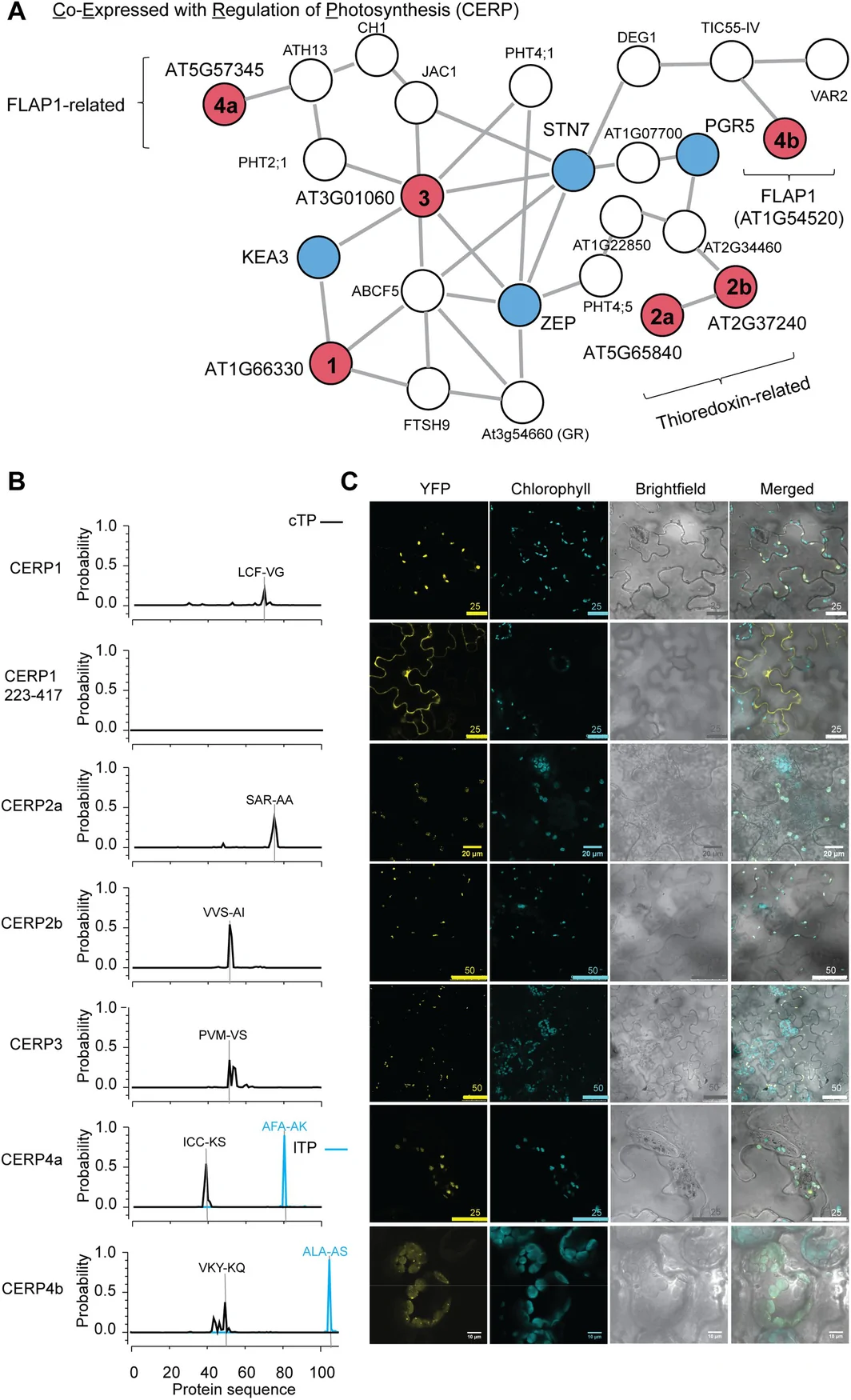
Selected co‐expressed with regulation of photosynthesis (CERP) proteins have predicted cTPs and localize to the chloroplast. (A) A co‐regulation network visualized with cytoscape of the photosynthetic regulators *STN7* (*AT1G68830*), *ZEP* (*AT5G67030*), *KEA3* (*AT4G04850*) and *PGR5* (*AT2G05620*) in blue circles, a number of genes with known and unknown functions as white circles (known described by their aliases) and selected genes with unknown or incomplete characterizations as red circles. (B) Cleavage probability of a chloroplast transit peptide (cTP) and lumen transit peptide (lTP) as predicted by TargetP for CERP1‐CERP4b. For CERP1, in addition to the full version, a truncated version was used that lacked a large part of the N‐terminus including the predicted cTP. (C) Localization of CERP‐YFP fusions by transient expression in leaves of *Nicotiana benthamiana* shows that all proteins localized to the chloroplast as predicted. Only the CERP1 truncation without a predicted cTP localized to the cytosol. Scale bar sizes are in microns.

Genes were selected either for their high number of network connections or for their similarity to already characterized regulators. These genes were designated *C*o‐*E*xpressed with *R*egulation of *P*hotosynthesis (CERP) 1–4 (Fig. 1A). CERP1 (AT1G66330) and CERP3 (AT3G01060) showed multiple connections within the network, while CERP2A and CERP2B encode two homologous thioredoxin‐like proteins (Fig. S1A). The protein encoded by CERP4A shows similarity to that of CERP4B (Fig. S1B), which is also present in the network and was previously described as important for plant acclimation to fluctuating light (Sato *et al*. 2017). All CERPs are conserved in the green lineage, except for CERP4a, which can only be found in flowering plants (Fig. S2). CERP2a and CERP2b, belonging to the superfamily of thioredoxin‐like proteins, have orthologues throughout eukaryotic life (Fig. S2).

The encoded proteins were analysed for their predicted localization using TargetP (Fig. 1B). All six proteins carried predicted chloroplast targeting peptides (cTPs) at their N‐termini. CERP4a and CERP4b were predicted to carry an additional lumen transit peptide (lTP). To confirm the localization, C‐terminal YFP fusions were transiently expressed in *N. benthamiana* leaves (Fig. 1C). For all full constructs, YFP fluorescence overlaid with the Chl *a* fluorescence, supporting that these fusion proteins indeed localize to the chloroplast. As a negative control, a CERP1 construct with an N‐terminal deletion was expressed, which showed localization in the cytosol.

### Mutants lacking CERP1 and CERP4b show increased NPQ under dynamic light conditions

**Fig. 2:**
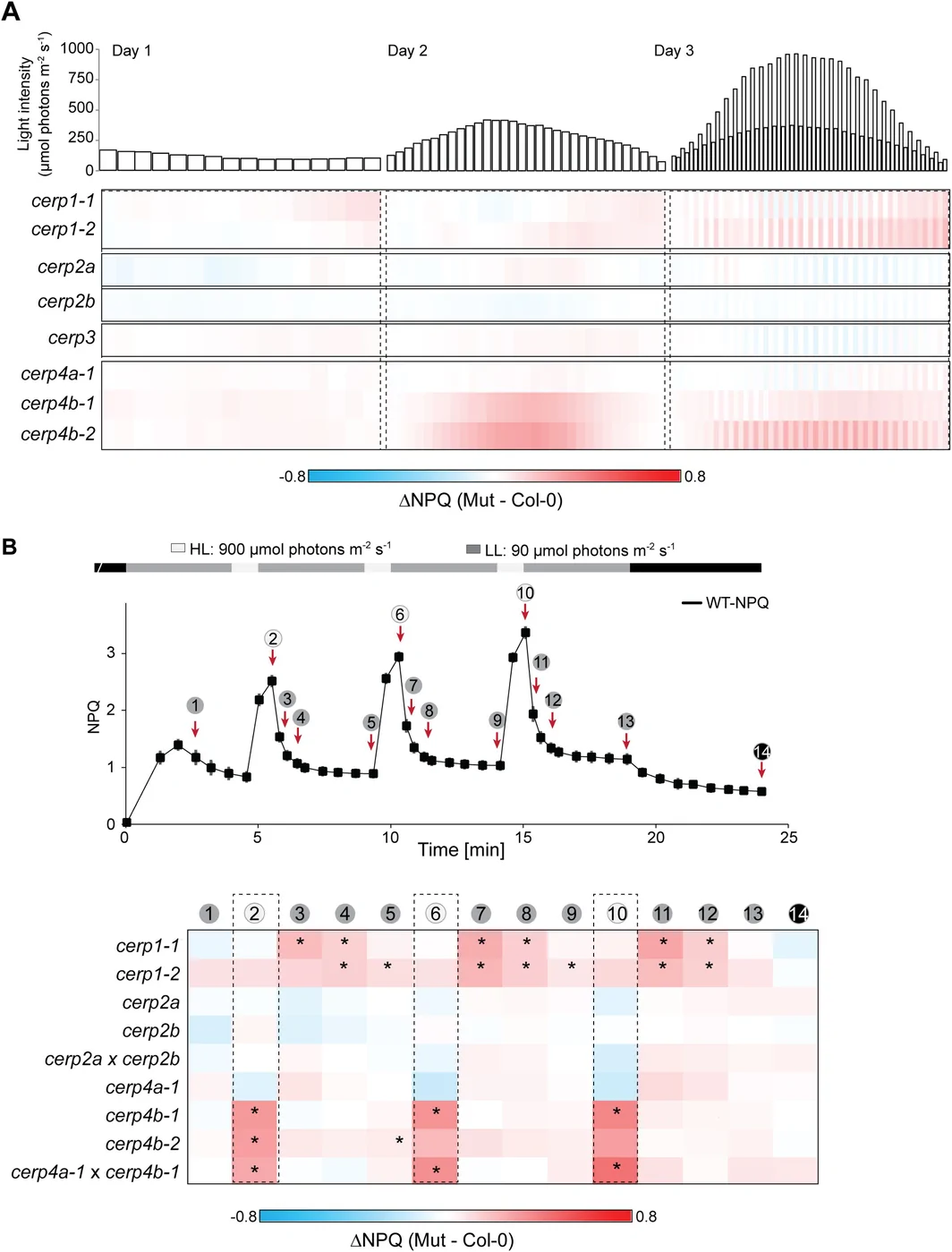
Lack of some CERPs results in NPQ differences in fluctuating light. (A) NPQ analyses of *cerp* mutants during three consecutive days of DEP Imaging, first with growth light, second with sinusoidal day and increased peak light intensity and third with fluctuations imposed on the sinusoidal day. (B) NPQ analyses of selected *cerp* single and double mutants during light fluctuations at time points indicated in the upper NPQ graph of WT (Col‐0). Data were analysed using one‐way ANOVA followed by Tukey's *post hoc* test. When data did not meet normality and homogeneity of variances criteria for ANOVA, a Kruskal–Wallis test followed by Dunn's *post hoc* test (with Bonferroni correction) was applied. Asterisks indicate significant differences to Col‐0. (A, B) Shown is average NPQ difference between mutants and WT in false colour.

T‐DNA insertion *cerp*‐mutant lines were confirmed by PCR and sequencing of the T‐DNA flanking regions (Tables S1 and S2) and analysed using the Dynamic Environmental Photosynthetic Imagers (DEPI) system under growth light conditions (day 1), a sinusoidal day with increased peak light intensities (day 2) and a sinusoidal day with superimposed higher light intensity phases (day 3) for their NPQ behaviour (Fig. 2A, Table S3). While few differences were observed between the mutants and the corresponding wild‐type (Col‐0) lines during growth light conditions (day 1), *cerp4b* mutants showed an increased NPQ during the higher light phases of the sinusoidal day 2 and an increased NPQ response during the high light phases of day 3. The *cerp1* mutants, instead, showed increased NPQ mainly during the lower light phases of day 3. While the *cerp2b* and *cerp4a* mutants showed values similar to Col‐0 throughout the experiments, slight differences were observed for *cerp2a* and *cerp3* on day 3. Here, both mutants showed mildly decreased NPQ during the high light phases of the light fluctuations.

To obtain a better time resolution, we measured NPQ during faster light fluctuations using an Imaging PAM. To understand whether the two proteins with sequence similarities have a redundant function, we generated double mutants of *cerp2a* and *cerp2b* and *cerp4a* and *cerp4b*. The NPQ analysis under fluctuating light intensities confirmed the DEPI results (Fig. 2B, Table S4, Fig. S3). Both *cerp1* mutants displayed significantly increased NPQ compared to Col‐0 after transition from high to low light. The increased NPQ fully relaxed within the 5‐min dark period at the end of the fluctuations, suggesting that it is due to higher qE. The *cerp4b* mutant showed increased NPQ values compared to Col‐0 particularly during the high light phase. Both *cerp4b* mutants showed significant increases in NPQ during the first high light phase of three consecutive fluctuations. The *cerp4a‐1 cerp4b‐1* double mutant resembled the *cerp4b‐1* single mutant with significantly increased NPQ during the high light phases compared with Col‐0. The NPQ of both *cerp2a* and *cerp2b* and their respective double mutant behaved very similar to Col‐0.

Taken together, these analyses revealed and confirmed NPQ phenotypes mainly of the *cerp1* and *cerp4b* mutants, particularly under fluctuating light conditions. While *cerp1* displayed a slowed NPQ relaxation, *cerp4b* showed enhanced NPQ, particularly in high light phases in line with previous findings (Sato *et al*. 2017). The results uncover that CERP1 modulates NPQ relaxation and support a function for CERP4b in NPQ suppression under high light.

### The *cerp1* and *cerp4b* mutants show contrasting differences in chlorophyll content and maximum quantum efficiency of PSII under both control and fluctuating light conditions

**Fig. 3:**
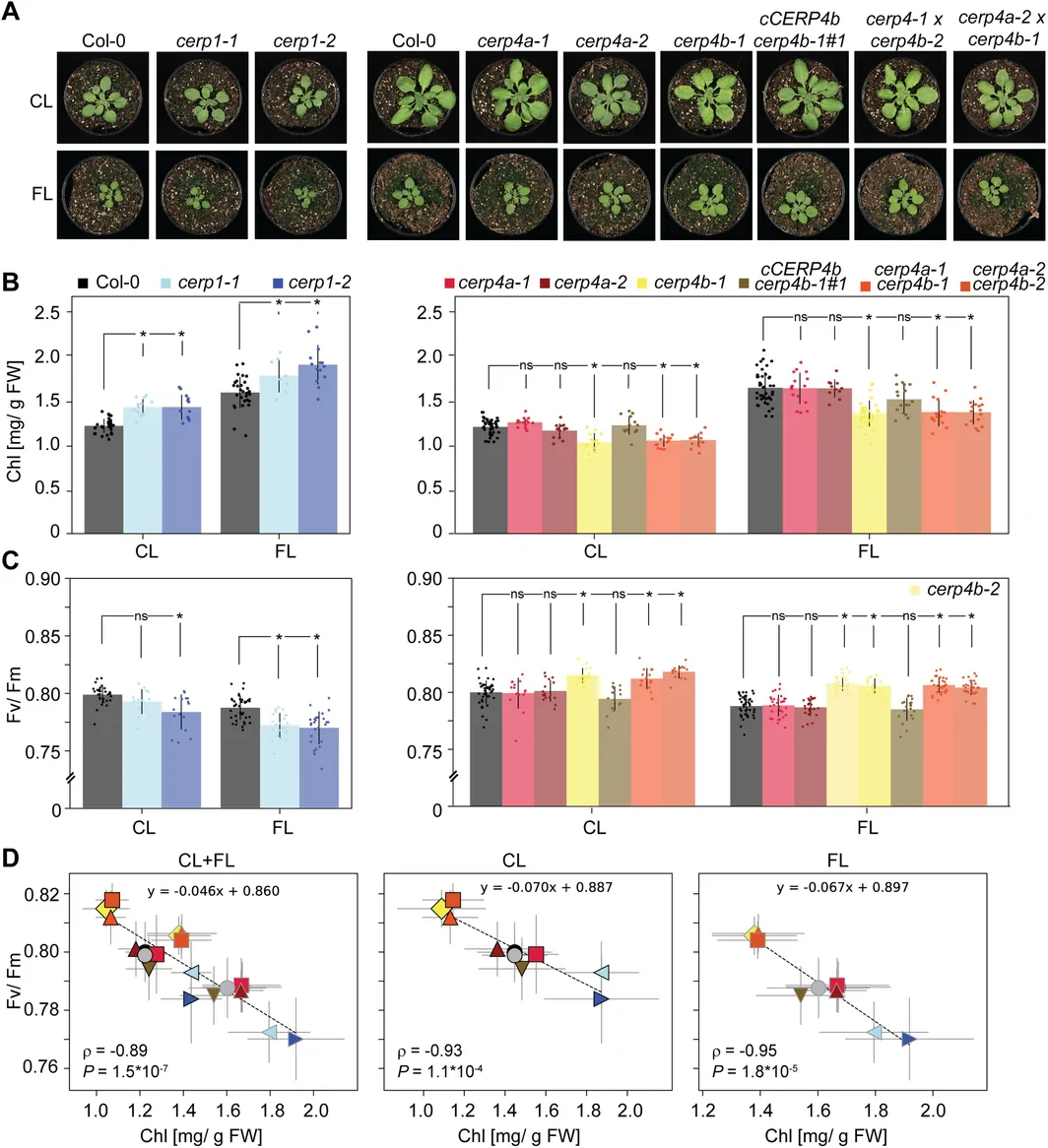
Lack of CERP1 and CERP4b results in opposite differences in chlorophyll content accompanied by inverse effects on F_v_/F_m_. (A) Pictures of 4‐week‐old Col‐0, *cerp1*, *cerp4a*, *cerp4b* single mutants, a *CERP4b* complementation (*cCERP4b*) line in the *cerp4b‐2* background and *cerp4a cerp4b* double mutants grown in control light (12 h day at 250 μmol photons m^−2^ s^−1^, upper panel) or fluctuating light (FL, light intensity of 90 μmol photons m^−2^ s^−1^ for 4 min, followed by 900 μmol photons m^−2^ s^−1^ for 1 min, lower panel). (B, C) Leaf chlorophyll content (B) and F_v_/F_m_ (C) of plants grown in CL and FL. Shown are single data points and averages ± SD. Data were analysed using one‐way ANOVA followed by Tukey's *post hoc* test. When data did not meet normality and homogeneity of variances criteria for ANOVA, a Kruskal–Wallis test followed by Dunn's *post hoc* test was applied. Asterisks indicate significant differences to Col‐0 (ns, not significant). (D) Analysis of mean Fv/Fm over chlorophyll content reveals a tight negative correlation with highly negative Spearman correlation coefficients (ρ) and low *P*‐values, particularly when calculated per each individual growth condition.

To further characterize *cerp1* and *cerp4* mutants, we grew plants under fluctuating light (FL) and non‐fluctuating control light (CL). As reported previously (von Bismarck *et al*. 2023), FL reduced overall growth (Fig. 3A), but no growth differences were observed between the mutants and Col‐0 under either condition. Because *cerp1* and *cerp4b* had reported differences in Chl content (Chen *et al*. 2011; Sato *et al*. 2017), we measured Chl per leaf fresh weight in 4‐week‐old plants from both conditions (Fig. 3B, Table S5). In general, Chl content was higher under FL than under CL. Consistent with earlier work, *cerp1* showed an increase, while *cerp4b* displayed a decrease in Chl relative to Col‐0; both differences were of similar magnitude and statistically significant in the two light conditions. For *cerp4b*, this was in contrast to previous reports, which showed decreases in Chl content to be much stronger under fluctuating light conditions (Sato *et al*. 2017). We also quantified Chl in *cerp4a* single and *cerp4a cerp4b* double mutants. As with the NPQ phenotype, *cerp4a* showed wild‐type levels of Chl and the double mutants showed a similar decrease in Chl content compared to Col‐0 as the *cerp4b‐1* single mutant.

The opposing changes in Chl content in the two mutants translated into contrasting effects on the maximum quantum yield of PSII (Fig. 3C, Table S5). The *cerp1* mutants displayed reduced F_v_/F_m_ values, which were significant for both alleles under FL conditions but not under CL, while *cerp4b* showed significantly higher Fv/Fm values under both conditions. The *cerp4a* mutant behaved like Col‐0, and the *cerp4axcerp4b* double mutant mirrored the *cerp4b* single mutant. In summary, the dataset further confirmed that lack of CERP4a does not exacerbate the *cerp4b* phenotype, implying that the two proteins are not functionally redundant; consequently, *cerp4a* single and *cerp4a cerp4b* double mutants were not pursued further.

Overexpression of the *CERP4b* coding sequence in the *cerp4b‐1* background restored both Chl content and Fv/Fm to wild‐type levels. Plotting Fv/Fm against Chl content revealed a strong negative correlation across all genotypes and growth regimes (Fig. 3D), indicating that the Fv/Fm differences are a direct consequence of altered Chl levels. This slope of the relationship was highly similar between FL‐grown and CL‐grown plants, but shifted towards higher Chl content in FL.

To further investigate whether the pigmentation phenotypes depended on growth light conditions, we also grew *cerp1* and *cerp4b* mutants under high light (HL, 900 μmol photons m^−2^ s^−1^) and determined Chl content in 4‐week‐old plants (Fig. S4). All genotypes displayed markedly lower Chl than under FL or CL. Under HL, only *cerp4b* differed significantly from Col‐0, showing the greatest chlorophyll reduction of all three light regimes (Fig. S4). By contrast, the *cerp1* mutants had only slightly and not significantly increased chlorophyll levels compared to Col‐0 in HL, indicating that their increased Chl content under lower light is conditional and largely disappears at higher growth light intensities.

### The *cerp1* mutants show specific differences in pigment composition particularly when grown in fluctuating light

**Fig. 4:**
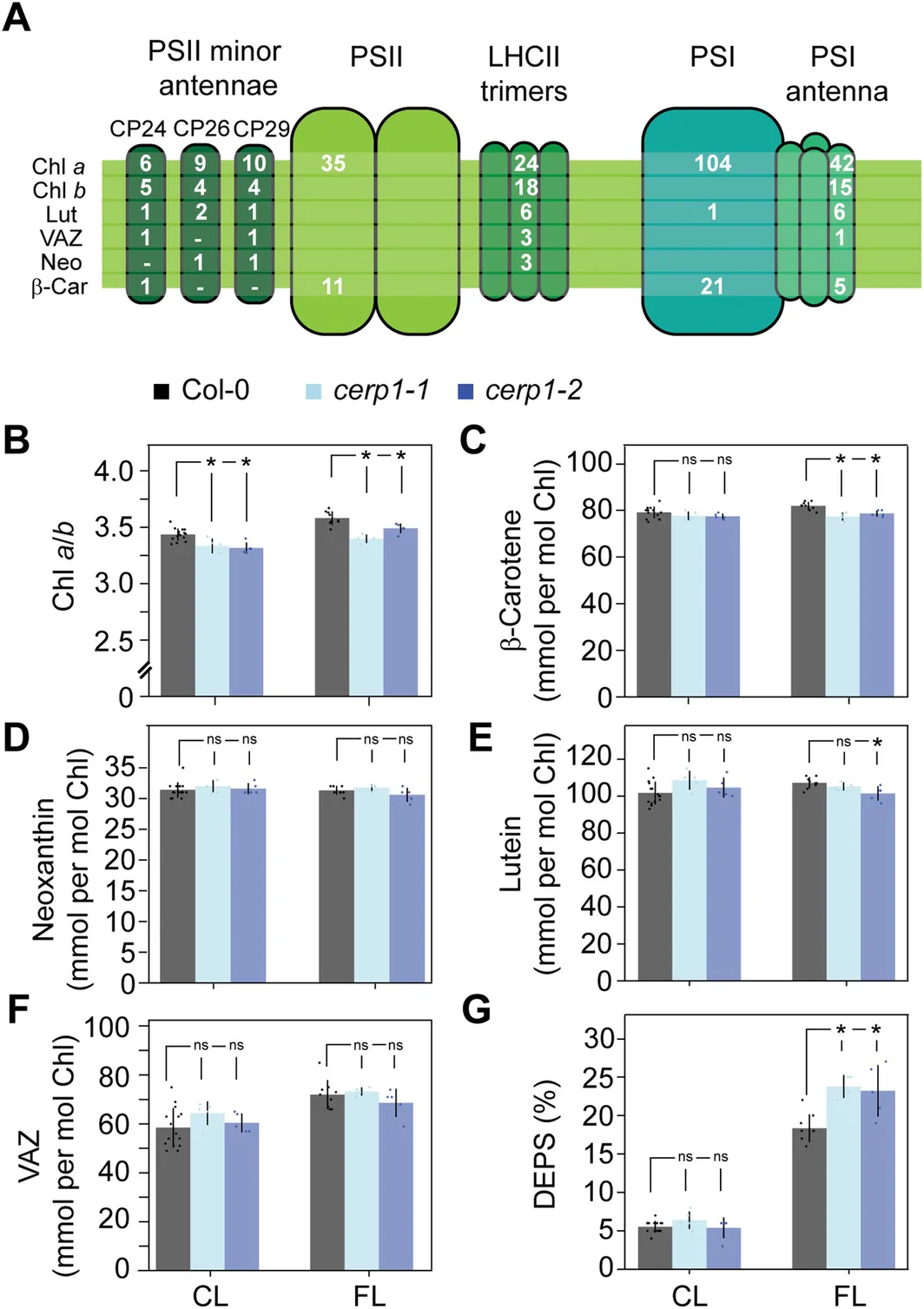
Plants lacking CERP1 show distinct changes in pigment composition. (A) Scheme depicting the distinct pigment compositions of the photosystems (PS) and their antennae (LHCII, light harvesting complex of PSII). (B–G) Leaves of 4‐week‐old Col‐0, *cerp1‐1*, *cerp1‐2* (grown in constant light, CL and fluctuating light, FL) were analysed for the chlorophyll *a/b* ratio (Chl *a/b*, B), β‐Carotene (C), neoxanthin (D), lutein (E) and VAZ pool (F) per total chlorophyll, as well as the xanthophyll deepoxidation state (DEPS, G). Shown are single data points and averages ± SD. Data were analysed using one‐way ANOVA followed by Tukey's *post hoc* test. When data did not meet the normality and homogeneity of variances criteria for ANOVA, a Kruskal–Wallis test followed by Dunn's *post hoc* test was applied. Asterisks indicate significant differences to Col‐0 (ns, not significant). The analysis was not corrected for multiple testing.

To further understand the increased Chl content phenotype of *cerp1*, plants grown in CL and FL were analysed for the content of other thylakoid pigments involved in light harvesting and photoprotection (scheme of photosynthetic complexes and their pigment content shown in Fig. 4A). The Chl *a/b* ratio was significantly lower in *cerp1* under both conditions, as compared to Col‐0 (Fig. 4B, Table S6). Differences in the Chl *a*/*b* ratio can be explained by variations in the stoichiometries of photosynthetic complexes, that is, changes in the ratio of PSII to PSI, or PSII antenna to PSII reaction centre. The carotenoid β‐carotene, which is mainly bound by the PSII and PSI reaction centres and the PSI antenna, but not the LHCII trimer, was slightly but significantly decreased per total chlorophyll in FL. The antenna pigments neoxanthin and lutein accumulated to WT levels, except for *cerp1‐2* under FL, which had slightly lower lutein per total chlorophyll (Fig. S4). There was no effect of the *cerp1* mutations on the pool of the violaxanthin, antheraxanthin and zeaxanthin (VAZ) under the measured conditions nor the de‐epoxidation state (DEPS) of the VAZ pool in CL (Fig. 4F,G). However, both *cerp1* mutant alleles showed significantly increased DEPS in FL.

While in CL the higher Chl content and lower Chl *a/b* ratio of *cerp1* mutants compared to Col‐0 were not accompanied by any significant differences in the content of other thylakoid pigments per chlorophyll, *cerp1* growing in FL had increased DEPS compared with Col‐0 and slightly lower ß‐carotene per total chlorophyll content.

### CERP1 is localized in the chloroplast stroma and CERP4b in the thylakoid membrane

**Fig. 5:**
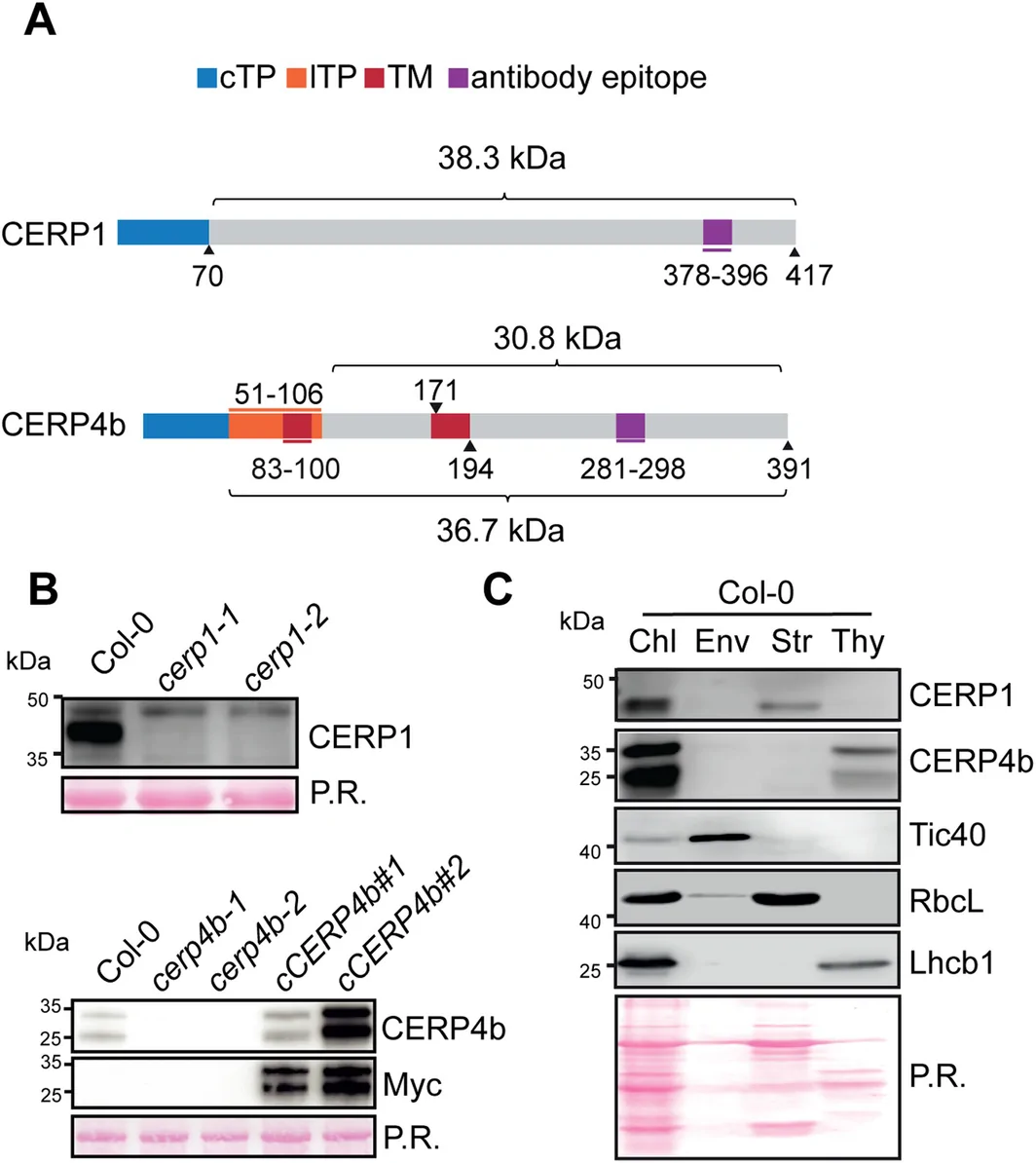
CERP1 and CERP4b are localized in the stroma and the thylakoid membrane, respectively. (A) Linear schemes of CERP1 and CERP4b proteins with lengths of targeting peptides, predicted transmembrane (TM) helices, epitope regions used for antibody generation in rabbits and predicted molecular weights. (B) Antibodies made against CERP1 and CERP4b recognize the respective proteins as evidenced by the lack of bands in the respective mutants. The double protein pattern is also detected by the Myc antibody in the *cCERP4b* lines in which a Myc‐tagged CERP4b protein complements the phenotype of the *cerp4b‐2* mutant. For control Ponceau Red (P.R.) stained RbcL is shown. (C) The fractionation of chloroplasts shows that CERP1 is localized in the stroma and CERP4b in the thylakoid membrane. For control, the nitrocellulose membrane prior to specific protein detection was stained with P.R. and envelope membranes were detected with antibodies against Tic40, stroma fraction with RbcL and thylakoids with Lhcb1.

CERP4b was previously reported to localize to chloroplast thylakoid and envelope membranes through detection of a Flag‐tagged CERP4b protein (Sato *et al*. 2017). To validate these results and characterize the sub‐chloroplastic localization of CERP1, epitope‐specific antibodies against both proteins were generated in rabbits (Fig. 5A). The specificity of both antibodies was tested on total leaf protein extracts of WT, mutants and in the case of CERP4b the complemented lines, which overexpressed the CERP4b protein (Fig. 5B, Fig. S5). For CERP1, we always detected one signal around 40 kDa in Col‐0, which was absent in both mutants. This was in line with the predicted molecular weight of mature CERP1 (‐cTP), namely 38.3 kDa. For CERP4b, we always detected a double band with fairly similar intensities in Col‐0, which was absent in both mutants. This double band was also found in the complementation lines and here also with the Myc antibody against a C‐terminal Myc tag. These immunoblot results strongly suggest that plant leaves carry two versions of the CERP4b protein with different molecular sizes. The CERP4b protein contains a predicted site for cleavage of a lumen targeting peptide (lTP). Thus, both CERP4b versions may vary in the presence of this lTP, with the fully processed version having a predicted molecular weight of 30.8 kDa and the partially processed, lTP carrying version of 36.7 kDa. These predicted sizes are in agreement with the detected signals by immunoblotting. The functional antibodies were then used on fractionated chloroplasts together with specific marker antibodies, Tic40 for the inner envelope membrane, RbcL for the stroma and Lhcb1 for the thylakoid membrane. While CERP1 was only found in the stromal fraction together with RbcL, both CERP4b versions were only detected in the thylakoid fraction together with Lhcb1 (Fig. 5C).

Taken together, the results show that CERP1 is a soluble protein located in the chloroplast stroma, while CERP4b occurs as two versions with varying sizes, the lower one of which may be explained by the cleavage of an lTP containing sequence during lumen import. Both CERP4b versions were located in thylakoid membrane fractions.

### Thylakoid complex composition remains largely unchanged in *cerp4b*

**Fig. 6:**
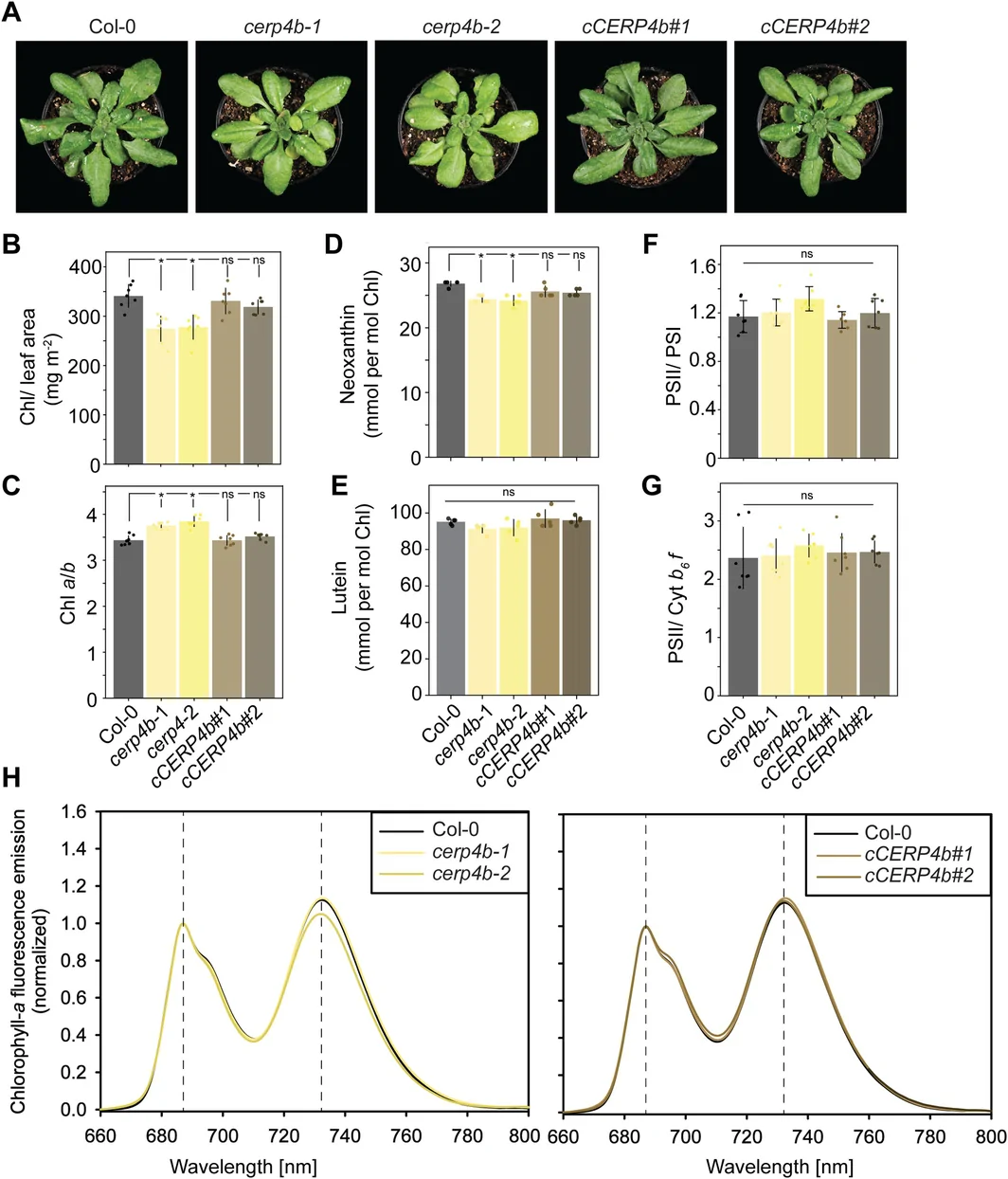
*cerp4b* mutants have lower chlorophyll content, a higher chlorophyll *a/b* ratio, but no measurable differences in photosynthetic complex composition. (A) Pictures of 5‐week‐old Col‐0, *cerp4b* mutants and complementation lines (*cCERP4b*) grown for 2 weeks in short days (8 h light, 16 h darkness) and then for 3 weeks in long days (16 h light, 8 h darkness), which show visibly paler leaves in the *cerp4b* mutants. (B, C) Leaf discs of mature leaves of plants as in (A) were analysed for chlorophyll content (B) and chlorophyll *a*/*b* ratio (C). (D, E) Leaf pigments were analysed by HPLC and antenna pigments neoxanthin (D) and lutein (E) are shown as relative to the total chlorophyll content. (F, G) Thylakoid membranes isolated from the entire rosettes were spectroscopically quantified for photosynthetic complex‐specific redox‐active components and ratios of PSII per PSI (F) and PSII per cytochrome *b*_*6*_ *f* (G) are shown. (B–G) Shown are single data points and averages ± SD (n = 7 for Col‐0, *cerp4b‐2* and cCERP4#2; n = 8 for *cerp4b‐1* and cCERP4b#1 for B, C, F, G and n = 5 for all genotypes in D, E, Figs. S7 and S8). Data were analysed using one‐way ANOVA followed by Tukey's *post hoc* test. When data did not meet the normality and homogeneity of variances criteria for ANOVA, a Kruskal–Wallis test followed by Dunn's *post hoc* test was applied. Asterisks indicate significant differences to Col‐0 (ns, not significant). (H) Low‐temperature (77 K) fluorescence emission spectra of thylakoid membranes. Emission maxima of PSII‐LHCII at 686 nm and of PSI‐LHCI at 733 nm are indicated by dotted lines. The emission maximum of PSII‐LHCII at 686 nm was normalized to 1. The average of N = 7 is shown.

Because, in our hands, growth in fluctuating light had no major effect on phenotypic differences between *cerp4b* mutants and Col‐0, we continued the characterization of *cerp4b* grown in our regular growth chambers. Visual inspection of 5‐week‐old plants from these growth conditions showed that both *cerp4b‐1* and *cerp4b‐2* mutants exhibited paler leaves compared to Col‐0 (Fig. 6A). Accordingly, 5‐week‐old *cerp4b* mutant leaves exhibited a significantly decreased Chl content (Fig. 6B) and an increase in Chl *a/b* (Fig. 6C). In the complementation lines, both values were similar to Col‐0. Further pigment analyses revealed that neoxanthin content per total chlorophyll was significantly decreased by ~10% in both mutants compared with Col‐0 and recovered in the complementation lines (Fig. 6D, Table S7). As neoxanthin is only bound by the PSII antenna (Fig. 4A), this result suggests a specific decrease in PSII antenna in *cerp4b*. Average levels of the general antenna pigment lutein per total chlorophyll were slightly decreased by ~3% in *cerp4b* compared to Col‐0, but the difference was not significant (Fig. 6E). The spectroscopy‐based quantification of photosynthetic complexes (Table S8) showed no significant difference in the ratios of PSII to PSI ratio (Fig. 6F) nor of PSII to cytochrome *b*_*6*_ *f* (Cyt *b*_*6*_ *f*, Fig. 6G). The quantification of the amount of complexes per leaf area, PSII, Cyt *b*_*6*_ *f*, as well as in the mobile lumen redox carrier protein plastocyanin (Fig. S6A–C) revealed no significant differences between genotypes. However, PSI content (Fig. S6D) was slightly decreased in both *cerp4b* mutants, which was significant in *cerp4b‐1* but not in *cerp4b‐2* compared to Col‐0. The activity of the chloroplast ATP synthase was approximated by the proton conductance of the thylakoid membrane (*g*_*H*_^+^; Takizawa *et al*. 2007). No significant differences between the different genotypes were observed (Fig. S6E). 77 K Chl a fluorescence measurements showed very similar emission spectra between the different genotypes, confirming that the photosynthetic complex composition is largely unchanged (Fig. 6H) and suggesting that any reductions in PSII antenna size are compensated for by down‐regulating PSI accumulation. That effects on PSII antenna size were only minor was further corroborated by immunoblot analyses of Lhcb proteins, which showed little differences explainable by genotype (Fig. S7). Additionally, light response curves of PSII and PSI‐related parameters were measured, which showed very similar values between genotypes, except for NPQ, which was only slightly higher in both mutant alleles at higher light intensities (Fig. S8). In summary, the decreased total Chl content, increased Chl *a/b* ratio, decreased neoxanthin per total chlorophyll, the largely unaltered accumulation of the photosystems and 77 K Chl a fluorescence emission point to a slight decrease in PSII antenna in the *cerp4b* mutant, which might be partly compensated for by the small reduction in PSI accumulation.

### CERP4b co‐migrates with PSII supercomplexes

**Fig. 7:**
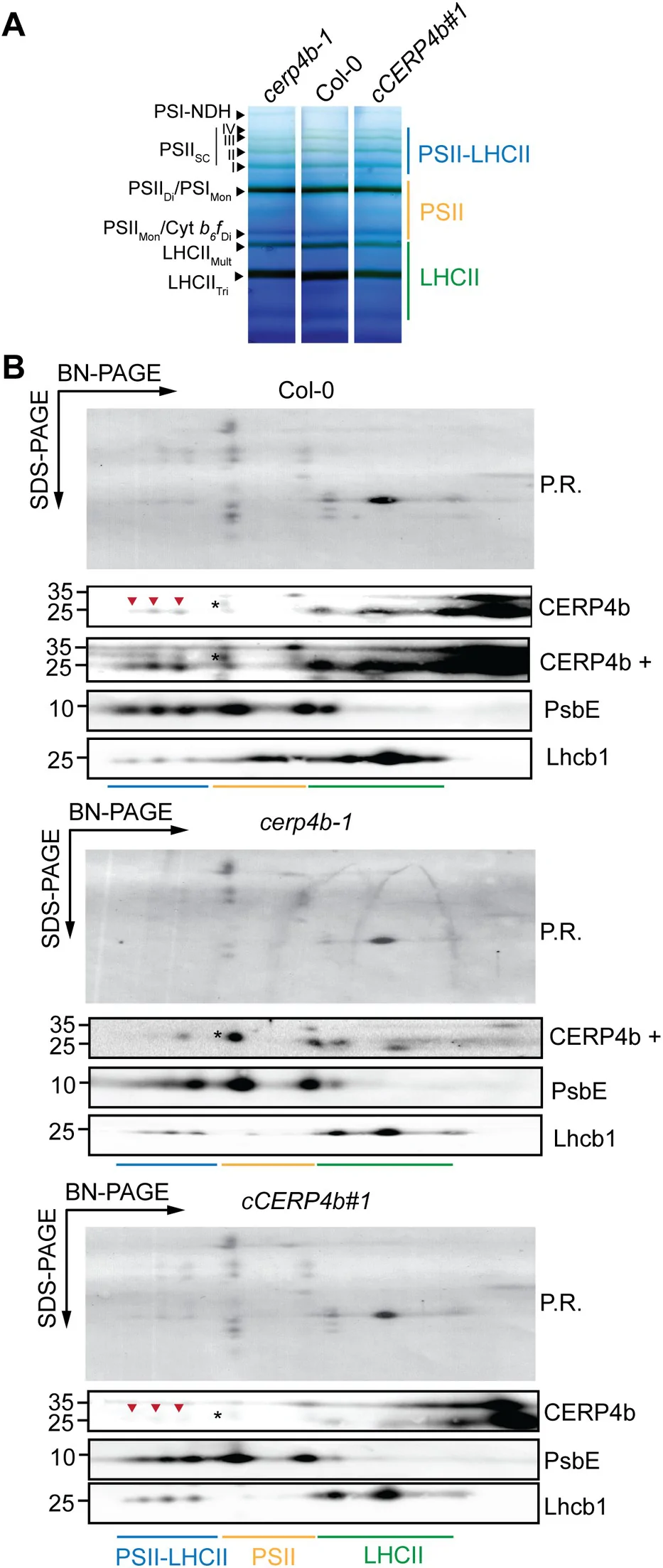
Processed CERP4b migrates with PSII supercomplexes. (A) Blue native (BN) PAGE analysis of *cerp4b‐1*, Col‐0 and a *CERP4b* complementation (cCERP4b) line shows lower accumulation of the highest molecular weight PSII supercomplex in *cerp4b‐1*. Visible protein complexes were assigned according to previous reports (Armbruster *et al*. 2014) and results shown below: photosystem (PS) I‐NAD(P)H dehydrogenase (NDH) complex (PSI‐NDH), PSII supercomplexes (PSII_SC_), PSI monomer and PSII dimer (PSI_Mon_/PSII_Di_), PSII monomer and dimeric cytochrome *b*_*6*_ *f* complex (PSII_Mon_/Cyt *b6f*_Di_), multimeric light‐harvesting complex (LHC) II (LHCII_Mult_), trimeric LHCII (LHCII_Tri_) and monomeric LHCII (LHCII_Mon_). (B) Second dimension immunoblot analyses using antibodies against the PSII core component PsbE (part of Cyt *b*_559_) and the Lhcb1 subunit of the PSII light harvesting major antenna, as well as CERP4b, reveal the smaller CERP4b version comigrates with PSII‐LHCII supercomplexes. The asterisk (*) indicates a major cross‐reaction of the CERP4b antibody with a protein in size in between the two CERP4b versions that co‐migrates with the PSII dimer/PSI monomer. The plus sign (+) specifies that the immunoblots were overexposed.

To further investigate the function of CERP4b in the accumulation of PSII antenna, we performed BN‐PAGE analysis of isolated thylakoids of Col‐0, the *cerp4b‐1* mutant and the complementation line #1 (*cCERP4b#1*), each corresponding to the same amount of Chl (Fig. 7A). In the first dimension, the thylakoid complex accumulation was largely similar between the different genotypes. The only difference was a decrease in the intensity of the highest PSII supercomplex in *cerp4b* as compared to Col‐0 and the complementation line. Complexes were then separated into their subunits by a second dimension and probed for CERP4b, as well as Lhcb1 and PsbE as markers for LHCII and the PSII reaction centre, respectively. In Col‐0 and *cCERP4b#1*, the two differently sized CERP4b versions were clearly detectable, mainly in the monomer and small complex fractions. In the *cerp4b‐1* mutant and Col‐0, the CERP4b signal was overexposed (+) and yielded an unspecific signal migrating at the PSII dimer/PSI monomer size in between the molecular weights of the larger and smaller CERP4b variants (in Col‐0). Interestingly, in the second dimensions of Col‐0 and *cCERP4b#1*, we found some of the smaller CERP4b version co‐migrating together with Lhcb1 and PsbE as part of the PSII supercomplexes, suggesting that upon processing, the CERP4b protein attaches to the PSII supercomplex. The BN results thus point to the possibility that the function of CERP4b in thylakoid pigment accumulation and composition is linked to its interaction with the PSII supercomplex.

## DISCUSSION

### The *cerp* mutants with stronger variations in NPQ under variable light show specific differences in chlorophyll content

We set out to investigate the function of genes belonging to a co‐expression network that includes key regulators of photosynthesis, such as KEA3, ZEP, STN7 and PGR5. We hypothesized that these genes co‐expressed with photosynthetic regulation (*CERP*), and additionally conserved across photosynthetic organisms, may themselves play roles in regulating photosynthesis. To test this, we examined whether corresponding mutants displayed phenotypes under variable light conditions. Our analysis focused on hub genes within this network (*e.g*., CERP1/AAF and CERP3) as well as genes whose products share similarities with proteins of known regulatory function, such as thioredoxin‐like proteins (CERP2 and CERP2b) and CERP4a, which shows similarity to CERP4b (also known as FLAP1).

All analysed CERPs localized to the chloroplast, consistent with the presence of a predicted cTP (Fig. 1) and in line with previous findings on CERP1/AAF and CERP4b/FLAP1 (Chen *et al*. 2011; Sato *et al*. 2017). When examining NPQ responses under fluctuating light conditions, we detected significant differences relative to the wild‐type Col‐0 only in *cerp1* and *cerp4b* (Fig. 2). While *cerp1* exhibited delayed NPQ relaxation in fluctuating light, *cerp4b* showed elevated NPQ during high light phases. Both mutants have been characterized previously, and we discuss our new observations in the context of earlier findings below. Notably, both mutants also displayed pronounced changes in Chl content, with *cerp1* showing increased and *cerp4b* decreased leaf Chl levels (Fig. 3). The difference in Chl content was accompanied by changes in the maximum quantum efficiency of PSII (Fv/Fm). The *cerp1* mutant with higher Chl content had lower Fv/Fm, while the *cerp4b* mutant with lower Chl content had higher Fv/Fm. The differences in pigment composition and Fv/Fm suggest that both mutants are affected in the function of their PSII antenna. Smaller antenna cross sections result in faster trapping of excitation energy in the PSII reaction centre and lower radiative loss by Chl *a* fluorescence emission (Wientjes *et al*. 2013). Taken together, our results indicate that the identified co‐regulation network of photosynthetic regulators includes proteins that modulate photosynthetic dynamics and the maximum quantum efficiency of PSII, at least in part through effects on the PSII antenna.

### CERP1 accelerates NPQ relaxation in fluctuating light

CERP1 was previously named Arabidopsis A‐15 (AAF), because of its homology to sweet potato A15 (SPA15), whose expression was strongly increased in senescing leaves (Chen *et al*. 2012). Fluorescence microscopy of an AAF‐GFP (CERP1‐GFP) fusion revealed its chloroplast localization, and loss‐of‐function mutants displayed higher Chl contents, whereas over‐expression lines contained less Chl. Although several experiments linked AAF to leaf senescence, its precise molecular role remained unresolved. In our study, *cerp1* mutants exhibited a slower relaxation of non‐photochemical quenching (NPQ, Fig. 2) after high light phases and a higher xanthophyll DEPS when grown under FL (Fig. 4). Because increased DEPS (*i.e*., elevated zeaxanthin) is known to slow down NPQ relaxation (Nilkens *et al*. 2010; von Bismarck *et al*. 2023), the mutant phenotype could, in part, be a consequence of altered zeaxanthin accumulation. However, when grown in CL, *cerp1* mutants showed wild‐type DEPS levels yet still displayed slowed NPQ relaxation after a single high light fluctuation. This observation indicates that CERP1 also influences NPQ dynamics independently of the initial DEPS. When grown in FL (but not in CL), *cerp1* mutants also showed a more complex rearrangement of thylakoid pigments: in addition to increased DEPS, carotenoid content relative to chlorophyll was reduced (Fig. 4). Since both wild‐type and *cerp1* plants are markedly smaller under FL than in CL, these FL‐specific pigment changes cannot be ascribed to premature senescence. Rather, they suggest that CERP1 integrates multiple environmental and developmental cues, including fluctuating light conditions, to coordinate the accumulation of Chl. In this context, CERP1 may regulate Chl synthesis or degradation, or more generally coordinate the balanced accumulation of chlorophylls and xanthophylls. Notably, the difference in Chl content between *cerp1* and Col‐0 was attenuated when plants were grown under high light (HL, Fig. S3), reinforcing the idea that CERP1's function is especially critical under fluctuating light regimes. Our chloroplast fractionation experiments placed CERP1 in the stromal compartment of chloroplasts, implying that its regulatory activity occurs in the soluble phase. Future work will aim to uncover the molecular mechanism by which stromal CERP1 modulates thylakoid pigment composition and NPQ relaxation.

### CERP4b modulates chlorophyll content and pigment composition and co‐migrates with PSII supercomplexes

CERP4b was originally designated FLAP1 (fluctuating‐light acclimation protein 1) because mutants displayed pronounced bleaching when grown under fluctuating light (Sato *et al*. 2017). These mutants showed higher NPQ, and a double mutant with *pgr5* partially suppressed lethality of *pgr5* in fluctuating light (Sato *et al*. 2017; Trinh *et al*. 2019). Consequently, CERP4b/FLAP1 was proposed to contribute to chloroplast proton homeostasis, possibly by regulating proton translocation across the thylakoid membrane. Some further supporting evidence for this notion came from experiments in cyanobacteria that used mutants in the homologue FlpA and showed attenuated extrusion of protons into the surrounding medium (Inago *et al*. 2020).

Our results instead point to a more direct role for CERP4b/FLAP1 in determining the size and/or pigment composition of the PSII antenna within the PSII supercomplex. First, *cerp4b/flap1* mutants contained less total chlorophyll and showed an increased Chl *a/b* ratio relative to Col‐0 across all growth light conditions with the most severe chlorophyll loss occurring under HL (Fig. 3B; Fig. S3). The elevated Chl a*/b* ratio was accompanied by a significant ~10% reduction in the PSII‐antenna‐specific pigment neoxanthin per total chlorophyll (Fig. 6B), suggesting that the mutants possess a slightly smaller PSII antenna than the wild type. Second, the *cerp4b‐1* mutant accumulated lower amounts of the largest PSII supercomplex (Fig. 7A). This supercomplex (C2S2M2) contains four LHCII trimers (Su *et al*. 2017). The reduction in C2S2M2 is consistent with a slight decrease in PSII antenna and implies that CERP4b/FLAP1 may influence either assembly or stability of this complex. Notably, mutants with reduced Chl b also accumulate less C_2_S_2_M_2_ (Friedland *et al*. 2019), so the observed decline could be a secondary effect of the lower Chl b content in *cerp4b/flap1* plants. Third, we show that the smaller CERP4b variant co‐migrates with PSII supercomplexes on BN second dimensions.

How can previous reports and our new data be consolidated? The PSII antenna is the main contributor to the fast NPQ component qE (Nicol *et al*. 2019). Thus, the changes in PSII antenna in the *cerp4b/flap1* mutants may directly affect qE amplitude independently of lumen pH. The partial rescue of the *pgr5* phenotypes in the *flap1 pgr5* double mutant is more difficult to reconcile. A decrease in PSII activity can suppress *pgr5* lethality in fluctuating light (Penzler *et al*. 2024), and thus, the partial survival of *flap1pgr5* mutants may also be due to a decrease in PSII activity. Indeed, double mutants showed a transient decrease in PSII quantum efficiency compared to *pgr5* when shifted from dark to low light (Trinh *et al*. 2019), which may be seen in support of such an idea. Definitive conclusions will require detailed analyses of pigment composition, PSII stability and functional parameters in both single and double mutants.

Although our data indicate changes in PSII antenna size or pigment load, *cerp4b/flap1* showed no measurable growth retardation under any of the tested conditions and no differences in the linear electron transport rate (Fig. 6B; Fig. S7). A minor reduction in PSI content was observed in the *cerp4b* mutants compared to Col‐0 (Fig. S6), which may be the result of an acclimatory response to balance excitation rates of both photosystems for efficient linear electron transport, as illustrated by the largely unchanged 77 K Chl *a* fluorescence emission spectra (Fig. 6H) and the unaltered light response curve of qL, a measure for the redox state of the PSII acceptor side (Fig. S9C). qL responds very sensitively to any imbalances in the excitation rates of both photosystems (*e.g*., Petersen *et al*. 2011; Rolo *et al*. 2024). The strong overexpression of *CERP4b* in one of the complemented mutant lines (cCERP4b#2; Fig. 5B) did not cause any differences in Chl and photosynthetic complex accumulation compared with Col‐0 (Fig. 6, Fig. S6). Also, light response curves of Chl *a* fluorescence and PSI‐related parameters (Fig. S8) were WT‐like in the overexpressor, suggesting that excess levels of CERP4b do not affect photosynthetic function.

A recent co‐immunoprecipitation (Co‐IP) analysis revealed that CERP4b/FLAP1 interacts with VIPP1, a protein involved in thylakoid biogenesis and PSII assembly (Yilmazer *et al*. 2025). While this suggests a joint role for VIPP1 and CERP4b/FLAP1, VIPP1 did not co‐precipitate LHCII or PSII core proteins, providing no direct evidence that the interaction between CERP4b/FLAP1 and VIPP1 influences antenna or core assembly. Using a CERP4b‐specific antibody, we identified two thylakoid‐localized variants: a lower‐molecular‐weight form that co‐migrates with PSII supercomplexes, and a higher‐molecular‐weight form that migrates with low‐molecular‐weight complexes (Fig. 5B). The size difference between these two variants is consistent with the presence or absence of an N‐terminal lumen‐targeting peptide, implying distinct sub‐chloroplastic localizations and possibly divergent functions. Accordingly, the lower‐molecular‐weight, lumen‐targeted isoform that co‐migrates with PSII supercomplexes may directly promote their assembly, stability or pigment loading, whereas the larger isoform could mediate the interaction with VIPP1. Future work employing complementation analyses and Co‐IP experiments with modified CERP4b/FLAP1 variants, either lacking the lTP cleavage site or fused to a more efficiently cleaved lTP will clarify whether the two versions carry out independent roles.

## CONCLUSIONS

The network analysis uncovered two chloroplast‐localized regulators, CERP1/AAF and CERP4b/FLAP, that modulate leaf pigment content and composition. Our data challenge previously reported roles for these two proteins but further investigations are needed to assign them distinct molecular functions. CERP1/AAF fine‐tunes chlorophyll accumulation and speeds up NPQ relaxation. An important unresolved issue is whether the acceleration of NPQ relaxation is mediated indirectly via changes in lumen pH or directly through alterations of the PSII antenna. Regarding CERP4b/FLAP, we found that the lumen‐targeted isoform co‐migrates with the PSII supercomplex, a finding that suggests, but does not prove, a physical interaction. Further experiments are required to validate a physical interaction of CERP4b/FLAP1 with the PSII supercomplex and to elucidate the functional interaction of CERP4b/FLAP1 with VIPP1.

## AUTHOR CONTRIBUTIONS

UA designed the project and performed co‐expression analysis. AZ and FD localized YFP‐tagged proteins. FD, EK, TB and VCG identified and characterized T‐DNA insertion lines. DEPI data were analysed by DMK. BAM generated *CERP4b* overexpression lines, validated CERP‐specific antibodies and performed further localization studies. PJ analysed pigments. WT and MAS quantified thylakoid complexes and performed light response curves. JMK performed BN‐analyses and immunoblot analyses. UA, JMK, EK, MAS and PJ interpreted data. UA wrote the manuscript. All authors read and approved the final version of the manuscript.

## Supporting information


**Fig. S1.** Alignment showing similarities of CERP2 and CERP4 proteins. Protein sequences of CERP2a and b (A) and CERP4a and b (B) were aligned using Clustal‐Omega. Similarity shading was performed using Color Align Conservation of bioinformatics.org. The chloroplast targeting peptide (cTP, green) and lumen targeting peptide (lTP, olive) were predicted using Target‐P. The bold underlined K (lysine) indicates the N‐terminal amino acid after cleavage of the predicted cTP and the underlined sequence AK and AA for CERP4a and CERP4b, respectively, the N‐terminal amino acids after cleavage of the predicted lTP. Black and grey shading show identity and similarity of amino acids, respectively.
**Fig. S2**. Phylogenetic trees of the *CERP* genes. Phylogenetic trees were built using PhyloGenes analysing the *CERP* genes of *A. thaliana* (in red) against multiple species across the green lineage (*Amborella trichopoda, Brassica napus, Chlamydomonas reinhardtii, Glycine max, Klebsormidium nitens, Marchantia polymorpha, Oryza sativa, Physcomitrium patens, Selaginella moellendorffii*) and non‐plant organisms (*CERP2*). Note that *CERP4a* and *CERP4b* genes are not conserved enough to be recognized as paralogues by the PhyloGene program. The number of paralog genes identified by PhyloGenes per organism may differ by other programmes.
**Fig. S3**. NPQ traces of Col‐0 and cerp mutants as summarized by the heatmap in Fig. 2B. NPQ was determined during light fluctuations of WT and *cerp* mutants. For *cerp1*, traces of WT (Col‐0) and mutant progenies of the same heterozygous T2 plant are shown. For *cerp4*, results for Col‐0 were pooled, but results of single measurements of the individual plant lines are shown in Table S4. For *cerp2*, a Col‐0 from an independent seed stock was used.
**Fig. S4**. Growth in high light further lowers chlorophyll content in *cerp4b*, while differences in *cerp1* become less evident. Leaf chlorophyll content of 4‐week‐old Col‐0, *cerp1‐1*, *cerp1‐2* and *cerp4b* plants grown in high light (900 μmol photons m^−2^ s^−1^) in a 12‐h day period. Shown are single data points and averages ± SD. Data met the assumptions of normality and homogeneity of variances and thus were analysed using one‐way ANOVA followed by Tukey's *post hoc* test. Asterisks indicate significant differences to Col‐0 (ns, not significant).
**Fig. S5**. Testing of monospecific antibodies on Col‐0 and *cerp1* and *cerp4b* mutants. Shown are the initial blots of the functional monospecific antibodies on total protein extract from WT (Col‐0) and *cerp1* and *cerp4b* mutants that had been selected from the initial T2 seeds supplied by the stock centre as indicated at the top. The antibodies showed some cross‐reactivity as indicated by the asterisks. The red arrow indicates the specific signals, which are present in Col‐0, but absent in the relevant mutants.
**Fig. S6**. Spectroscopic complex quantification per leaf area. (A–D) Spectroscopic complex quantifications were performed on thylakoid preparations of 5‐week‐old Col‐0, *cerp4b‐1*, *cerp4b‐2*, *cCERP4b#1* and *cCERP4b#2*, for photosystem II (PSII, A), Cytochrome *b*_*6*_ *f* (Cyt *b*_*6*_ *f*, B), plastocyanin (PC, C) and photosystem I (PSI, D). All contents were then normalized to a leaf area basis. (E) The proton conductivity of the ATP‐synthase (*g*H^+^) was determined for the same set of plants before thylakoids were isolated. (A–E) Shown are single data points and averages ± SD (n = 7 for Col‐0, *cerp4b‐2* and cCERP4#2; n = 8 for *cerp4b‐1* and cCERP4b#1). Data were analysed using one‐way ANOVA followed by Tukey's *post hoc* test. When data did not meet normality and homogeneity of variances criteria for ANOVA, a Kruskal–Wallis test followed by Dunn's *post hoc* test was applied. Asterisks indicate significant differences to Col‐0 (ns, not significant). The analysis not corrected for multiple testing.
**Fig. S7**. Immunoblot analysis of Lhcb proteins. Immunoblot analysis of total protein extract from Col‐0 (gradient from 0.2 to 1.0), *cerp4b* mutants and *cCERP4b#1* using specific antibodies against Actin as loading control and light harvesting complex II proteins (Lhcb1, Lhcb2, Lhcb3, Lhcb4, Lhcb6). Ponceau red (P.R.) staining of one membrane before blotting is shown.
**Fig. S8**. Light response curves. (A–F), Light response curves of Chl *a* fluorescence and P_700_ difference absorbance parameters were performed on five‐week‐old Col‐0, *cerp4b‐1*, *cerp4b‐2*, *cCERP4b#1* and *cCERP4b#2* and electron transport rate at photosystem II (ETRII, A), non‐photochemical quenching (NPQ, B), the redox state of the PSII acceptor side (qL, C), the non‐regulated dissipation of excitation energy (Y(NO), D), donor side limitation of photosystem I (Y(ND), E) and its acceptor side limitation (Y(NA), F) are shown. For the donor‐side limitation, the first value indicates the maximum oxidation level of P_700_ reached by far‐red illumination in darkness Shown are averages of N = 21 ± SD.

**Table S1.** Primers used in this study.
**Table S2**. T‐DNA insertion mutants used in this work and the confirmed flanking sequence of the respective insertions.
**Table S3**. NPQ analysis of plants using the DEPI chamber.
**Table S4**. NPQ analysis of plants during light fluctuations using the Imaging‐PAM.
**Table S5**. Fv/Fm and chlorophyll content.
**Table S6**. Pigment content.
**Table S7**. Pigment content.
**Table S8**. Thylakoid complex content and ATP‐synthase conductivity.

## Data Availability

The data that supports the findings of this study are available in the Figs. S1–S8 and Tables S1–S8 of this article.
