# Supplementary material for: Two proteins co‐expressed with photosynthetic regulators affect photosynthetic dynamics linked to changes in thylakoid pigment content and composition
Source: Plant Biol (Stuttg). 2026 Jul 20;28(6):1796–812. doi: 10.1111/plb.70266 (PMC13586636; doi:10.1111/plb.70266)
Supplement: Supplementary file 1 [file PLB-28-1796-s001.docx]

**Two proteins co-expressed with photosynthetic regulators affect photosynthetic dynamics linked to changes in thylakoid pigment content and composition**

Jakob-Maximilian Keller^1^, Firdevs Dinc^1^, Elias Kaiser^1^, Arkadiusz Zupok^1^, Bethan A. Morris^1^, Thekla von Bismarck^1,2^, Viviana Correa Galvis^1^, Wolfram Thiele^1^, David M. Kramer^2,3^, Peter Jahns^4^, Mark A. Schöttler^1^ and Ute Armbruster^5,6^

**Supplemental Material**

**Supplemental Fig. 1 Alignment showing similarities of CERP2 and CERP4 proteins**

Protein sequences of CERP2a and b (A) and CERP4a and b (B) were aligned using Clustal-Omega. Similarity shading was performed using Color Align Conservation of bioinformatics.org. The chloroplast targeting peptide (cTP, green) and lumen targeting peptide (lTP, olive) were predicted using Target-P. The bold underlined K (lysine) indicates the N-terminal amino acid after cleavage of the predicted cTP and the underlined sequence AK and AA for CERP4a and CERP4b respectively, the N-terminal amino acids after cleavage of the predicted lTP. Black and grey shading show identity and similarity of amino acids, respectively.

**Supplemental Fig. 2 Phylogenetic trees of the *CERP* genes**

Phylogenetic trees were built using PhyloGenes analyzing the *CERP* genes of *A. thaliana* (in red) against multiple species across the green lineage (*Amborella trichopoda, Brassica napus, Chlamydomonas reinhardtii, Glycine max, Klebsormidium nitens, Marchantia polymorpha, Oryza sativa, Physcomitrium patens, Selaginella moellendorffii*) and non-plant organisms (*CERP2*). Note that *CERP4a* and *CERP4b* genes are not conserved enough to be recognized as paralogues by the PhyloGene program. The number of paralog genes identified by PhyloGenes per organism may differ by other programs.

**Supplemental Fig. 3 NPQ traces of Col-0 and cerp mutants as summarized by the heatmap in Fig. 2B**

NPQ was determined during light fluctuations of WT and *cerp* mutants. For *cerp1*, traces of WT (Col-0) and mutant progenies of the same heterozygous T2 plant are shown. For *cerp4*, results for Col-0 were pooled, but results of single measurements of the individual plant lines are shown in Supplemental Fig. S4. For *cerp2*, a Col-0 from an independent seed stock was used.

**Supplemental Fig. 4 Growth in high light further lowers chlorophyll content in *cerp4b*, while differences in *cerp1* become less evident**

Leaf chlorophyll content of 4-week-old Col-0, *cerp1-1*, *cerp1-2* and *cerp4b* plants grown in high light (900 µmol photons m^-2^ s^-1^) in a 12 h day period. Shown are single data points and averages ± SD. Data met the assumptions of normality and homogeneity of variances and thus were analyzed using one-way ANOVA followed by Tukey’s post hoc test. Asterisks indicate significant differences to Col-0 (ns, not significant).

**Supplemental Fig. 5 Testing of monospecific antibodies on Col-0 and *cerp1* and *cerp4b* mutants**

Shown are the initial blots of the functional monospecific antibodies on total protein extract from WT (Col-0) and *cerp1* and *cerp4b* mutants that had been selected from the initial T2 seeds supplied by the stock center as indicated at the top. The antibodies showed some cross-reactivity as indicated by the asterisks. The red arrow indicates the specific signals, which are present in Col-0, but absent in the relevant mutants.

**Supplemental Fig. 6 Spectroscopic complex quantification per leaf area**

(A-D) Spectroscopic complex quantifications were performed on thylakoid preparations of five-week-old Col-0, *cerp4b-1*, *cerp4b-2*, *cCERP4b#1* and *cCERP4b#2*, for photosystem II (PSII, A), Cytochrome *b_6_f* (Cyt *b_6_f*, B), plastocyanin (PC, C), photosystem I (PSI, D). All contents were then normalized to a leaf area basis. (E) The proton conductivity of the ATP-synthase (*g*H^+^) was determined for the same set of plants before thylakoids were isolated. (A-E) Shown are single data points and averages ± SD (n = 7 for Col-0, *cerp4b-2* and cCERP4#2; n = 8 for *cerp4b-1* and cCERP4b#1). Data were analyzed using one-way ANOVA followed by Tukey’s post hoc test. When data did not meet normality and homogeneity of variances criteria for ANOVA, a Kruskal–Wallis test followed by Dunn’s post hoc test was applied. Asterisks indicate significant differences to Col-0 (ns, not significant). The analysis not corrected for multiple testing.

**Supplemental Fig. 7 Immunoblot analysis of Lhcb proteins**

Immunoblot analysis of total protein extract from Col-0 (gradient from 0.2 to 1.0), *cerp4b* mutants and *cCERP4b#1* using specific antibodies against Actin as loading control and light harvesting complex II proteins (Lhcb1, Lhcb2, Lhcb3, Lhcb4, Lhcb6). Ponceau red (P.R.) staining of one membrane before blotting is shown.

**Supplemental Fig. 8 Light response curves**

(A-F), Light response curves of Chl *a* fluorescence and P_700_ difference absorbance parameters were performed on five-week-old Col-0, *cerp4b-1*, *cerp4b-2*, *cCERP4b#1* and *cCERP4b#2* and electron transport rate at photosystem II (ETRII, A), non-photochemical quenching (NPQ, B), the redox state of the PSII acceptor side (qL, C), the non-regulated dissipation of excitation energy (Y(NO), D), donor side limitation of photosystem I (Y(ND), E) and its acceptor side limitation (Y(NA), F) are shown. For the donor-side limitation, the first value indicates the maximum oxidation level of P_700_ reached by far-red illumination in darkness Shown are averages of N= 21 ± SD.
